# Atypical presentation of *TRPV3* variant: Cerebral palsy and intellectual disability without dermatologic features of olmsted syndrome

**DOI:** 10.1016/j.jdcr.2024.10.038

**Published:** 2024-12-25

**Authors:** Mohamed Adil Shah Khoodoruth, Widaad Nuzhah Chut-kai Khoodoruth, Yasser Saeed Khan

**Affiliations:** a Child and Adolescent Mental Health Service, Hamad Medical Corporation, Doha, Qatar; b Division of Genomics and Precision Medicine, College of Health and Life Sciences, Hamad Bin Khalifa University, Doha, Qatar; c Division of Child & Adolescent Psychiatry, Department of Psychiatry, Schulich School of Medicine and Dentistry, Western University, London, Ontario, Canada; d Centre of Disease Control and Prevention Department, Hamad Medical Corporation, Doha, Qatar; e College of Medicine, Qatar University, Doha, Qatar

*To the Editor:* Olmsted syndrome (OS) is a rare genodermatosis, with limited cases reported globally, primarily characterized by progressive palmoplantar keratoderma and keratotic plaques, and is linked to mutations in the *TRPV3* gene on chromosome 17p13.2.^1^^,^^2^ We read with great interest the case report by Frantz et al, which describes a mild presentation of OS due to a loss-of-function mutation in the *TRPV3* gene (transient receptor potential vanilloid-3).^3^ Their patient exhibited dermatologic features, including nonpruritic hyperkeratotic palms and soles with scarring alopecia.

Here, we report a 17-year-old male of Asian descent, with cerebral palsy and intellectual disability, but without any dermatologic features typically associated with OS, despite carrying a *TRPV3* variant (Fig 1). He was referred for psychiatric evaluation due to academic decline, impulsivity, and emotional dysregulation. A physical exam demonstrated no evidence of palmoplantar keratoderma, periorificial plaques, or any other skin lesions. Cognitive testing revealed an FSIQ of 69 (95% confidence interval, 65-76), indicating intellectual disability. Genome-wide oligonucleotide array-based comparative genomic hybridization analysis, performed using the Human Genome CGH Microarray kit, identified an intragenic deletion of approximately 13 kilobases (kb) in the *TRPV3* gene at cytogenetic band 17p13.2. The deleted genomic segment included exons 3-8 of the TRPV3 gene (arr[GRCh38] 17p13.2(3532389_3545489)×1). Since parental genetic data were unavailable, it remains unclear whether this variant was inherited or de novo.

**Fig 1:**
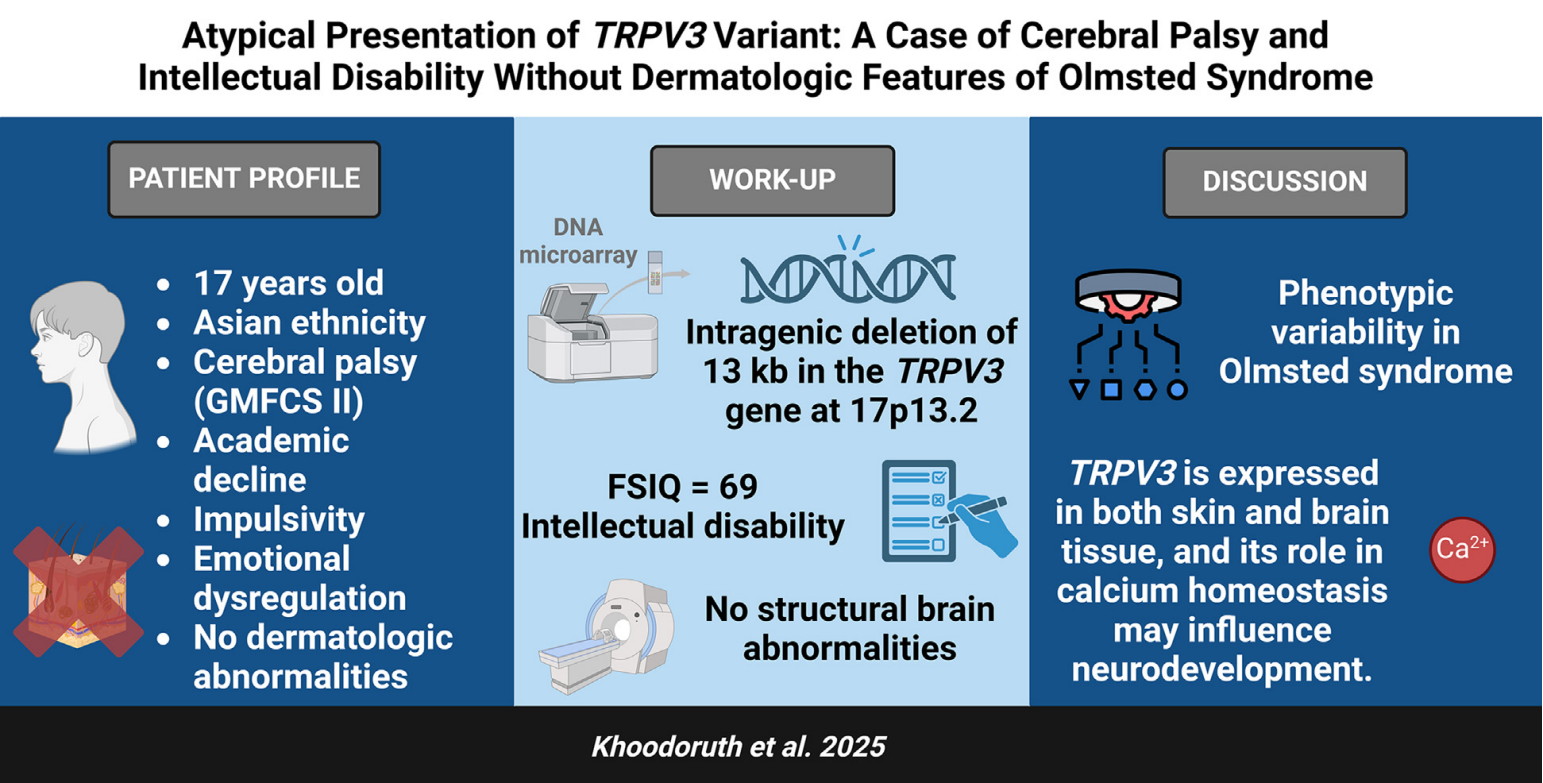
Profile of the patient.

This case highlights the phenotypic variability associated with *TRPV3* variants. Our case expands this understanding by demonstrating that *TRPV3* variants may also be associated with significant neurological symptoms in the absence of dermatologic abnormalities. In fact, the original patient with OS exhibited psychomotor retardation and cognitive impairment, highlighting the possibility of neurological involvement in some individuals.^4^ While TRPV3's role in skin biology is well established, its expression in the brain indicates a potential involvement in neurodevelopmental disorders, likely through its role in regulating calcium homeostasis.^5^

In short, the phenotypic variability in our patient highlights that *TRPV3* variants can cause both neurological and dermatological symptoms. However, the absence of dermatologic signs in our case underscores the need for individualized diagnosis and suggests *TRPV3*'s role in neurodevelopment. Our case expands the understanding of *TRPV3* variants, emphasizing the importance of considering genetic testing in patients with intellectual disability and cerebral palsy, even without skin manifestations.

## Conflicts of interest

None disclosed.
